# Reconstruction of the Core and Extended Regulons of Global Transcription Factors

**DOI:** 10.1371/journal.pgen.1001027

**Published:** 2010-07-22

**Authors:** Yann S. Dufour, Patricia J. Kiley, Timothy J. Donohue

**Affiliations:** 1 Department of Bacteriology, University of Wisconsin – Madison, Madison, Wisconsin, United States of America; 2 BACTER Institute, University of Wisconsin – Madison, Madison, Wisconsin, United States of America; 3 Department of Biomolecular Chemistry, University of Wisconsin – Madison, Madison, Wisconsin, United States of America; Stanford University, United States of America

## Abstract

The processes underlying the evolution of regulatory networks are unclear. To address this question, we used a comparative genomics approach that takes advantage of the large number of sequenced bacterial genomes to predict conserved and variable members of transcriptional regulatory networks across phylogenetically related organisms. Specifically, we developed a computational method to predict the conserved regulons of transcription factors across α-proteobacteria. We focused on the CRP/FNR super-family of transcription factors because it contains several well-characterized members, such as FNR, FixK, and DNR. While FNR, FixK, and DNR are each proposed to regulate different aspects of anaerobic metabolism, they are predicted to recognize very similar DNA target sequences, and they occur in various combinations among individual α-proteobacterial species. In this study, the composition of the respective FNR, FixK, or DNR conserved regulons across 87 α-proteobacterial species was predicted by comparing the phylogenetic profiles of the regulators with the profiles of putative target genes. The utility of our predictions was evaluated by experimentally characterizing the FnrL regulon (a FNR-type regulator) in the α-proteobacterium *Rhodobacter sphaeroides*. Our results show that this approach correctly predicted many regulon members, provided new insights into the biological functions of the respective regulons for these regulators, and suggested models for the evolution of the corresponding transcriptional networks. Our findings also predict that, at least for the FNR-type regulators, there is a core set of target genes conserved across many species. In addition, the members of the so-called extended regulons for the FNR-type regulators vary even among closely related species, possibly reflecting species-specific adaptation to environmental and other factors. The comparative genomics approach we developed is readily applicable to other regulatory networks.

## Author Summary

An important property of living systems is the use of regulatory networks to appropriately program gene expression. Central to the function of regulatory networks are transcription factors that regulate gene expression by binding to specific DNA sequences. Despite the central role of these regulatory networks, the processes driving their organization and evolution across organisms are poorly understood. This paper describes the use of comparative genomics and high-throughput approaches to predict the organization and evolution of transcriptional regulatory networks across a large group of species. We focused on regulatory networks controlling cellular responses to changes in O_2_ levels because this signal has major consequences on many biological systems. Our analysis predicts that related regulatory networks share a core set of target genes across diverse species while other target genes vary according to the organism's specific lifestyle. Our approach of defining transcriptional regulatory networks across a wide range of organisms should be of general utility to studying similar questions in other systems.

## Introduction

Organisms rely on regulatory networks to integrate and process signals from various sources and to orchestrate the transcription of genes controlling a range of cellular processes. Importantly, changes in the architecture of these regulatory networks play a significant role in survival or adaptation of organisms to changing environments [1]. However, the processes underlying regulatory network evolution across related organisms are unclear. To address this problem we used a comparative genomics approach that takes advantage of the large number of sequenced bacterial genomes to predict the architecture and infer the evolutionary history of regulatory pathways controlling the biological response to O_2_.

Fundamental to the function of transcriptional regulatory networks are DNA-binding proteins that recognize specific DNA target sequences to modulate gene expression. Accordingly, identifying the set of target genes for each transcription factor is a crucial step toward understanding the functions of their target genes, elucidating the architecture of regulatory networks, and inferring how these networks have evolved. Yet, the set of target genes recognized by a given transcription factor is rarely available for a regulator within or across species. Because related organisms often rely on orthologous regulators for similar functions, comparative genomics approaches offer the possibility to characterize regulons that are widely conserved across organisms, as well as to identify important exceptions. In this study, we used computational and high-throughput experimental methods to predict the members of transcriptional regulatory networks that are conserved across a large number of diverse bacteria.

Our analysis focused on transcriptional regulatory networks that are known or predicted to function under low O_2_ or anaerobic conditions. These transcriptional regulatory networks are often conserved across species because the availability of O_2_ has major consequences for many critical metabolic activities. For example, in bacteria, O_2_ availability controls the type of energetic pathway used for growth (fermentation, respiration, photosynthesis in anaerobic phototrophic bacteria, etc.) and acquisition of nutrients (nitrogen or carbon dioxide fixation, or metal uptake, etc.), which are critical to the survival of cells, communities, and entire ecosystems [2]. While the physiological effects of O_2_ on these processes are fairly well established, the transcription factors, target genes, or regulatory networks controlling these functions are not as well understood. Consequently, information on the properties of these regulatory networks is necessary in order to identify conserved functions that are controlled by O_2_ availability across related organisms.

FNR, FixK, and DNR are related, and relatively well-studied members of the CRP/FNR super-family of transcription factors that control anaerobic processes in many proteobacteria [3]. FNR is a global regulator of anaerobic gene expression in *Escherichia coli* and its activity is directly inhibited by O_2_ via destruction of a labile iron-sulfur cluster [2], [4]. FNR orthologs are widely distributed across bacteria [3], but, to date, their function has been mostly studied in *E. coli* and other γ-proteobacteria [5]–[7]. FixK is another member of the CRP/FNR super-family that controls gene expression in an O_2_-dependent manner [8]. For example, in the α-proteobacterium *Bradyrhizobium japonicum* FixK_2_ plays a role in establishing the legume root-nodule symbiosis that occurs at low O_2_ tensions [8]. However, unlike FNR, the activity of FixK_2_ is not directly controlled by O_2_. Instead, *fixK_2_* expression is controlled by the O_2_-responsive two-component signal transduction system FixLJ [9]. Finally, DNR, another member of the CRP/FNR super-family, controls the expression of genes needed for anaerobic denitrification in *Pseudomonas aeruginosa* [10]. DNR activity responds to nitric oxide (NO), an intermediate of denitrification [11].

While the functions of FNR, FixK, and DNR have been established in several model organisms, it is not clear whether these roles are conserved across other species. Homologs of FNR, FixK, and DNR are known or predicted to exist in a large number of diverse bacteria [3], but the target genes for these regulators have not been extensively studied. In addition, the fact that FNR, FixK, and DNR have significant amino acid sequence similarity in their DNA-binding domains and recognize very similar DNA target sequences [3] makes it challenging to predict their respective target genes. It also raises the question of how these functions can be selectively controlled in organisms that contain different numbers of one or all three of these proteins. Furthermore, because it is not possible to rely solely on the presence of a predicted upstream DNA target sequence as a means to link a target gene to the regulon of one of these transcription factors, it is difficult to predict the regulatory network or biological functions controlled by FNR, FixK, or DNR orthologs within or across different organisms using current approaches. These challenging properties illustrate why an approach integrating additional information is necessary to predict the regulatory networks of related proteins across organisms.

In this report, we describe a computational method that takes advantage of the large number of available bacterial genome sequences to predict the conserved portions of the respective regulons of related transcription factors. After clustering members of the CRP/FNR super-family into sets of orthologs, we predicted genes that are controlled by FNR, FixK, or DNR proteins by comparing the phylogenetic profiles of the regulators with the profiles of putative target genes. We chose to focus on α-proteobacteria since these species are metabolically diverse, have several unique anaerobic lifestyles (photosynthesis, symbiosis, nitrogen fixation, denitrification) when compared to organisms analyzed previously, and often contain multiple protein members of one or more of the CRP/FNR sub-families. To provide experimental support for the computational predictions, we defined genes in the *Rhodobacter sphaeroides* FnrL (a FNR-type regulator) regulon using a combination of chromatin immuno-precipitation on a chip (ChIP-chip) assays [12] and publically available transcription profiling data [13]–[16]. The results reported here refined predictions for the DNA target sequences of members of the CRP/FNR super-family and predicted conserved members of the FNR, FixK, and DNR regulons across α-proteobacteria. The patterns of regulon conservation observed across the α-proteobacteria phylogeny led us to propose that the regulon of each conserved regulator is composed of a core set of genes conserved across species. We also propose that this core regulon is expanded in each species by incorporating genes whose functions are selected by the conditions found in their ecological niches.

## Results

### The CRP/FNR super-family of α-proteobacteria is represented by 8 major conserved sub-families

Our approach to determining the members of the FNR, FixK, and DNR regulons across the α-proteobacteria was to first identify all the sub-families of the CRP/FNR super-family in α-proteobacteria and then predict their DNA target sequences. Phylogenetic analysis of the CRP/FNR super-family from bacteria in 2002 [3], revealed 21 distinct protein sub-families, which included FNR, FixK, and DNR. Because a larger number of α-proteobacterial genomes were available in 2009, we performed a similar analysis to determine the representation and distribution of these sub-families within the α-proteobacteria. In addition, a second goal was to determine whether any new sub-families share a similar predicted DNA target sequence to FNR, FixK, and DNR that would confound our analysis.

**Figure 1:**
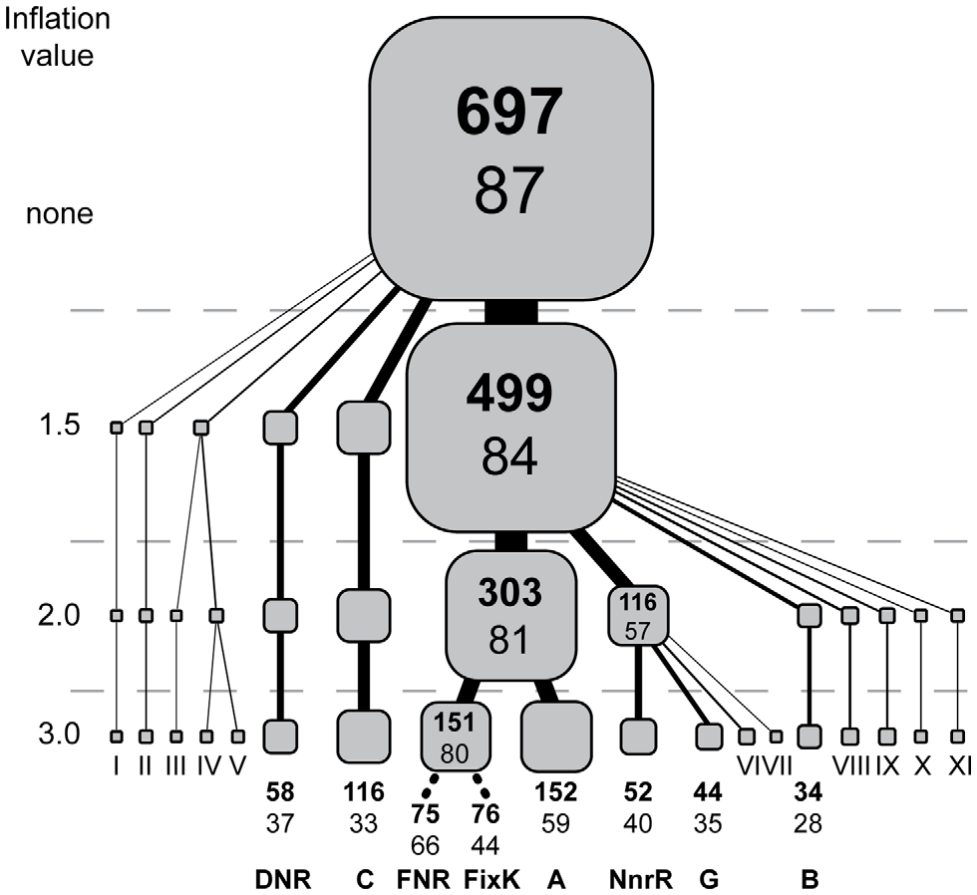
Major sub-families of the CRP/FNR-type transcription factors in 87 representative α-proteobacteria. The hierarchical tree representation of the amino acid sequence similarities was constructed by partitioning protein groups using increasing clustering stringency (inflation value, see Materials and Methods). The bold numbers within each box represent the number of individual proteins within each cluster and the number below represents the number of species possessing at least one of these proteins. The bottom of the tree shows names for the major 8 sub-families using nomenclature described previously [3]. Minor sub-families could not be classified definitively, so these sub-families are designated by roman numerals.

After searching all sequenced α-proteobacterial genomes in the Integrated Microbial Genomes database (img.jgi.doe.gov) in January 2009 (∼150 genome sequences) for proteins of the CRP/FNR super-family, we first found that α-proteobacteria from the genera *Rickettsia*, *Ehrlichia*, *Wolbachia*, and *Bartonella* do not possess proteins in the CRP/FNR super-family. Accordingly, these genera were not studied further. Among the remaining genera, we selected 87 representative α-proteobacterial species that altogether contained 697 proteins in the CRP/FNR super-family (Table S1). To assemble these 697 proteins into functionally related sets, we took a clustering approach derived from the ORTHOMCL algorithm [17], which identifies connected sets of proteins in networks constructed from protein sequence similarities. When we applied this clustering approach multiple times with increasing stringency, we uncovered a hierarchical relationship between proteins of the different families (Figure 1). Ultimately, 607 of the 697 proteins were clustered into 7 major sub-families that could not be further sub-divided solely by more stringent clustering, suggesting that the proteins within each of these 7 major sub-families are very closely related. The 7 α-proteobacterial protein families and their relationships are also consistent with the phylogenic tree obtained by neighbor joining of the 2002 dataset [3], supporting the conclusion that both approaches are capturing the same functional groups.

However, this approach failed to differentiate between FNR- and FixK-type proteins because it only considered global amino acid sequence similarities. Therefore, we subsequently divided the mixed FNR-FixK group into FNR or FixK groups based on the known properties of *E. coli* FNR. Specifically, *E. coli* FNR and presumably its orthologs have 4 conserved cysteine residues that are essential to coordinate an O_2_-labile [4Fe-4S] cluster [18], . Proteins within the mixed FNR-FixK group that lack any of the cysteine ligands for the [4Fe-4S] cluster are not expected to sense O_2_ directly and thus, were assigned to the FixK group. After sub-dividing the FNR-FixK group into a FNR group, members of which possess all 4 of the conserved cysteine residues, and a FixK group, which includes proteins that lack one or more of these 4 cysteines, 8 major protein sub-families of the CRP/FNR super-family were defined.

The resulting 8 major protein sub-families include members of the FNR, FixK, DNR, NnrR, A, B, C, and G groups of the CRP/FNR super-family using the nomenclature described by Korner *et al.* [3] (Figure 1; locus IDs for each sub-family are provided in Table S1). Our analysis indicates that only the 8 sub-families of the 21 sub-families of the CRP/FNR super-family identified across all species available in 2002 [3] are significantly conserved across the α-proteobacteria considered in our study, and no new conserved sub-families in addition to the 21 were identified. Proteins in the 11 remaining sub-families of the CRP/FNR super-family, such as CRP or CooA, were found in some of the 87 α-proteobacteria, but these other sub-families had a very limited distribution across species and clustered into minor groups (Figure 1).

### The 8 major sub-families of CRP/FNR super-family are unevenly distributed across the α-proteobacteria

The FNR sub-family is composed of 75 members distributed in 66 species and is the most widely distributed of the 8 sub-families of CRP/FNR super-family. The FixK sub-family comprises 76 proteins distributed in the genomes of 44 species. While members of the FixK sub-family are not predicted to sense O_2_ directly, the activity of some family members is indirectly regulated by O_2_ through the FixLJ two-component system [20]. However, we were unable to predict whether all the FixK orthologs were regulated by FixLJ since it is difficult to predict which species have FixLJ orthologs because functionally distinct two-component regulators have very similar amino acid sequences [21]. Nevertheless, FNR orthologs and some FixK orthologs are expected to regulate genes that have functions relevant to adapting to changes in O_2_ levels. The nitric oxide-responsive DNR and NnrR groups of regulators contain 58 proteins in 37 genomes and 52 proteins in 40 genomes, respectively. In contrast, the largest sub-family of proteins in α-proteobacteria is group A, which is composed of 152 uncharacterized proteins that are distributed in the genomes of 59 of the 87 species examined. The next largest sub-family, group C, contains 116 uncharacterized proteins that are distributed within the genomes of 33 species. Most of the species of α-proteobacteria, which possess a protein in group C, belong to the *Rhizobiales* order, suggesting that the proteins in this group are associated with a biological function that is conserved in these α-proteobacteria. The other two major groups, B (34 proteins in 28 genomes) and G (44 proteins in 35 genomes), are also composed of uncharacterized proteins. In summary, we predict that α-proteobacterial species possess different combinations of CRP/FNR-type regulators, including the FNR, FixK, and DNR families.

### Of the 8 major sub-families, only FNR, FixK, and DNR are predicted to recognize similar DNA sequences

**Figure 2:**
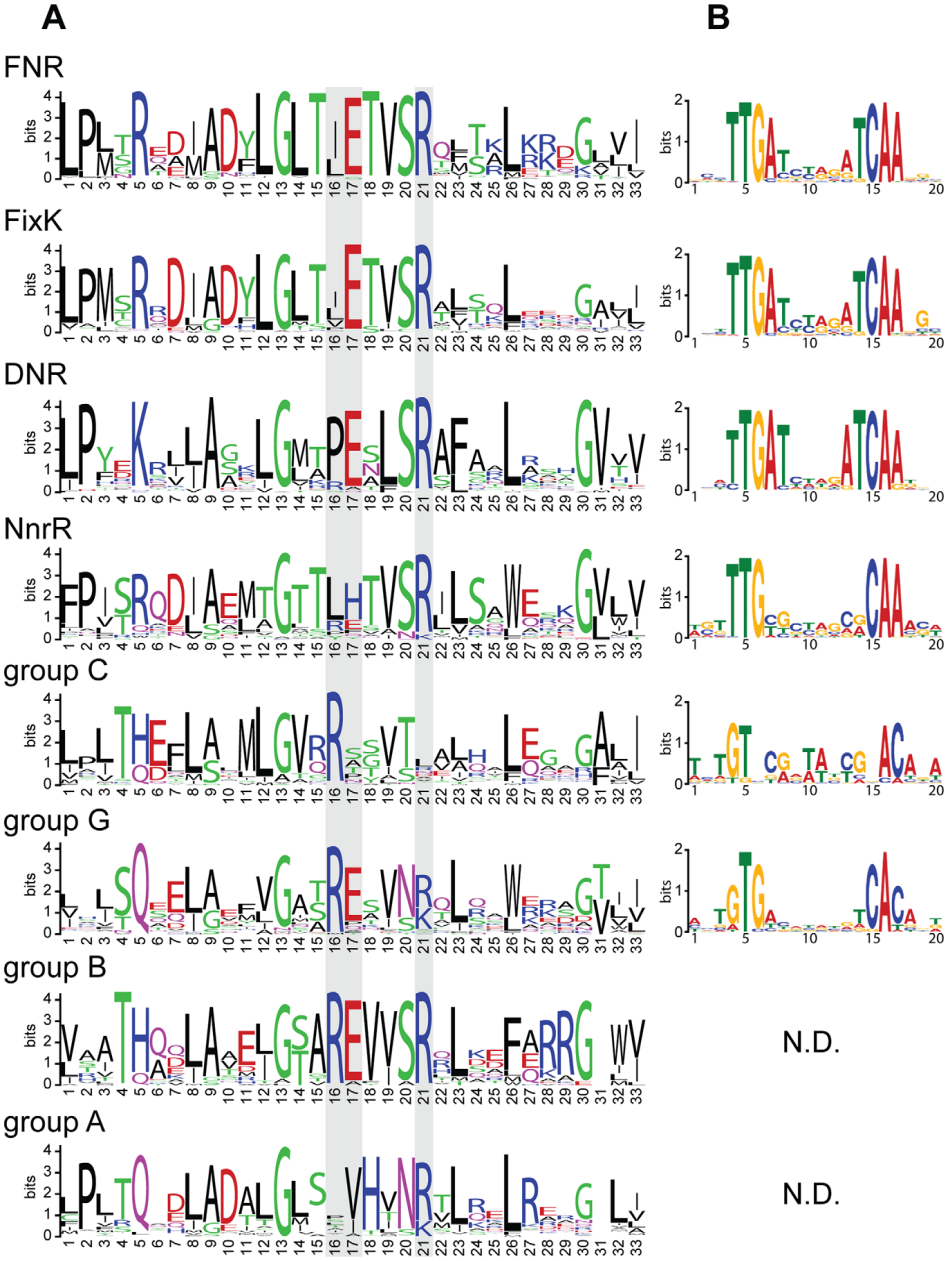
Protein binding domains and predicted DNA binding motifs of the CRP/FNR-type transcription regulators. (A) Logos representing the protein sequence alignments of the predicted helix-turn-helix domains of each of the 8 major sub-families of the CRP/FNR-type transcription regulators. The amino acid residues predicted from the *E. coli* CRP-DNA crystal structure to make base specific contacts [23] were mapped onto the sub-families and indicated with grey boxes. (B) Logos representing the predicted DNA binding motifs associated with each of the 8 major sub-families of the CRP/FNR-type transcription regulators. (N.D. means that a logo was not defined using criteria described in the text). For both (A,B), the heights of the letters represent their conservation (in bits) at a particular position in the multiple sequence alignments, and the numbers on the x-axis represent the relative position in the multiple sequence alignments.

Previous reports indicated that representative members of the FNR, FixK, and DNR families recognize similar DNA target sites [3], [8], [22], [23]. To determine, (i) if all proteins within and across each of the FNR, FixK, or DNR families share a conserved DNA target sequence and (ii) if any of the other 5 major sub-families of the CRP/FNR super-families also recognize similar sites, we analyzed amino acid sequences in the helix-turn-helix (HTH) DNA-binding domain within each sub-family. This information was then used to predict the corresponding DNA target sequences (Figure 2).

For this analysis, we first aligned the amino acid sequences of the C-terminal domains that contain the predicted HTH motif of the above set of 697 proteins. The multiple sequence alignment was then divided into the 8 respective sub-families. We also mapped onto the alignments the three corresponding residues of the *E. coli* CRP protein that make direct contact with DNA in the X-ray structure of the binary complex [24]. These CRP residues were used as a reference to assess conservation of the residues that would be predicted to determine DNA target sequence specificity between and within sub-families. This mapping revealed that two of the three corresponding residues in the FNR, FixK, and DNR sub-families are conserved across all members of these groups (glutamate and arginine at positions 17 and 21, respectively in Figure 2A). In addition, neighboring residues, which may directly or indirectly affect DNA target specificity, are also well conserved across these three protein families (positions 12, 13, 20 and 26 in Figure 2A). Furthermore, Glu 209 (position 17), Ser 212 (position 20), and Arg 213 (position 21) have all been implicated in specific DNA binding by *E. coli* FNR [25], [26]. The HTH domains of the remaining protein groups in the CRP/FNR super-family differ significantly from the ones of FNR, FixK, and DNR and from each other suggesting specialization of DNA binding (Figure 2A). In summary, even though the specific contribution of each residue to DNA target specificity is not totally understood for members of the FNR, FixK, and DNR families, the extensive conservation of amino acid residues in their HTH domains supports the hypothesis that proteins from these three sub-families, but not from the other 5 sub-families, recognize similar DNA target sequences.

To predict the corresponding DNA target sequences for proteins in the 8 CRP/FNR sub-families and to assess the predictions made from the HTH domain sequence analysis, we took advantage of the fact that transcription of genes encoding the proteins in the CRP/FNR super-family is often auto-regulated. Thus, we searched for conserved DNA sequences in the regions upstream of the structural genes in each group. The one exception to this approach was the FixK sub-family because transcription of *fixK* in *Bradyrhizobium japonicum*, and presumably orthologs in other species, are regulated by the response regulator FixJ. Indeed, a promoter sequence analysis revealed that only 20 of the 76 FixK orthologs may be auto-regulated. Thus, we derived the FixK binding motif from the promoter sequences of previously predicted targets genes in *B. japonicum* [8] and their orthologs in α-proteobacterial genomes containing orthologs of FixK. The results of this analysis showed that the predicted DNA target sequences for members of the FNR, DNR and FixK groups are virtually identical (Figure 2B). In contrast, analysis of upstream DNA sequences for the other 5 major groups of α-proteobacterial members of the CRP/FNR super-family indicated that these proteins bind related but non-identical target sites. For example, genes encoding proteins in the NnrR group are preceded by a DNA sequence that contains only 6 of the 10 conserved positions of the FNR, FixK, or DNR motifs (Figure 2B). This was not surprising since proteins in the NnrR group share less conserved residues with the FNR and FixK HTH domain (notably, residues at position 17 are different in Figure 2A). In conclusion, these findings reinforce the proposition that proteins in the FNR, FixK, and DNR groups recognize very similar, if not identical, DNA target sequences in the 87 selected α-proteobacteria.

### Predicting the regulon composition of FNR, FixK, and DNR across α-proteobacteria

Knowing the DNA target sequence for a transcription factor often provides sufficient information to predict computationally its target genes within and across genomes. However, using the deduced DNA binding sites to predict the respective FNR, FixK, and DNR regulons presented a particular challenge because the three regulators recognize very similar DNA target sequences (Figure 2B) and because the selected 87 α-proteobacterial species often possess different numbers or combinations of the FNR, FixK, and DNR proteins (Table S1). For example, *Rhodopseudomonas palustris* TIE-1 possesses three proteins representing each of the FNR, FixK, and DNR groups, while *Hoeflea phototrophica* DFL-43 has three proteins in the FNR group and none in the FixK, or DNR groups. Therefore, without additional information it was not possible to determine the respective regulons of FNR, FixK, and DNR by testing solely for the presence of a DNA target sequence that is common to these three regulators.

**Figure 3:**
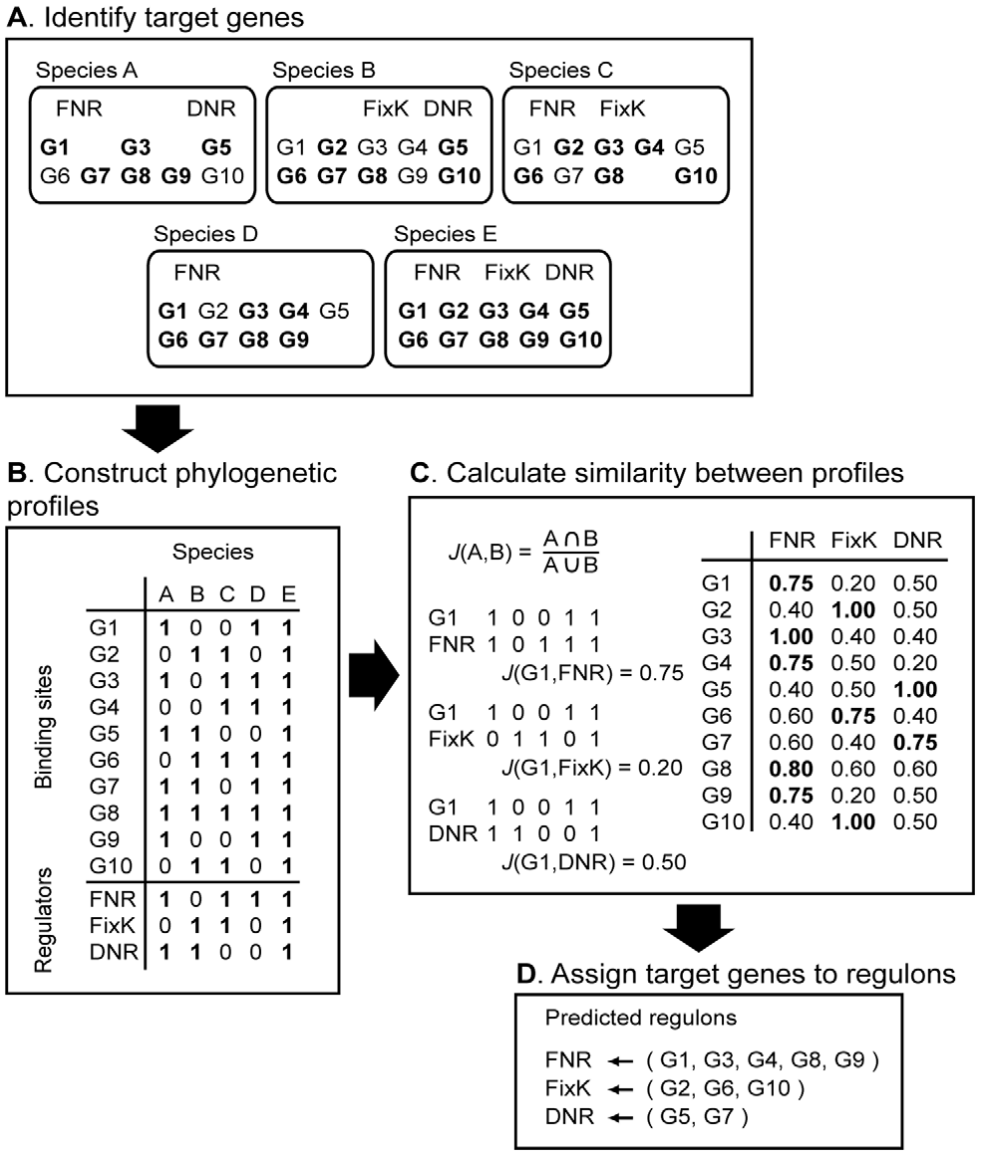
Schematic description of the stepwise prediction of the conserved regulons of FNR, FixK, and DNR. (A) The first step was to identify orthologous genes across species (G1, G2,…). Second, orthologous genes that also contain the target DNA-binding sequence in their promoter regions are indicated in bold. (B) Third, the phylogenetic profiles of the target genes found in sets of orthologous genes were constructed, and the phylogenetic profiles of the genes encoding for the transcription regulators. (C) Fourth, the similarities (*J*(A,B)) between each target gene profile and each regulator profile were calculated. (D) Finally, target genes were assigned to the regulator with which it shared the most similar phylogenetic profile.

However, if we assumed that the composition of regulons co-evolved with the function of their respective regulators, then we would expect that phylogenetic occurrence profiles across related species should contain information about the functional relationships between target genes and regulators. This information can then be used to assign target genes to their historical regulator even in situations where multiple regulators might have overlapping regulons. To characterize the evolutionary relationship between FNR, FixK, or DNR, and putative target genes, we improved upon a computational method that was used previously to predict regulon members of alternative sigma factors [12] by integrating it with an approach first introduced by Pellegrini *et al.*, who compared phylogenetic profiles of sets of orthologous genes across multiple species to infer functional links between genes [27] (Figure 3). Because the DNA target sequence of a particular regulator represents the functional link between the regulator and its target genes, we expected that the presence of the binding sequences in the promoter regions of the target genes to co-evolve also with the regulator function. Therefore, taking into account the correlation between the phylogenetic profiles of target genes and regulators should allow us to assign target genes to their historical regulators and define their respective core regulons even if the transcription factors have indistinguishable DNA-binding sequences. Note, we used the term “core regulon” to refer to a historical consensus that emerged from the comparison of the 87 bacteria considered in this study.

**Figure 4:**
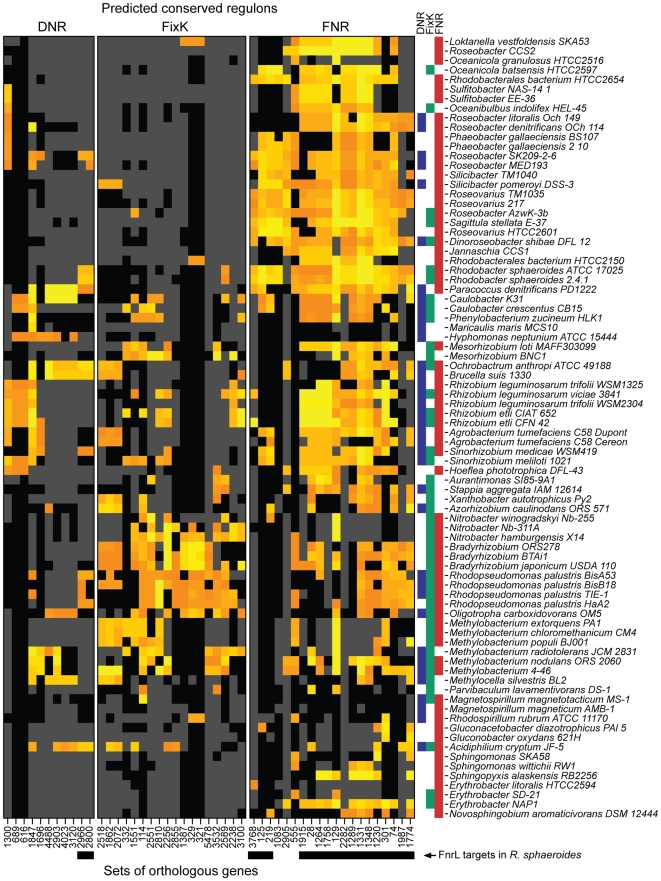
Predicted members of the DNR, FixK, and FNR regulons. The heatmap indicates whether the promoter region of the corresponding gene contains a significant match to the DNA target sequence shared by FNR, FixK, and DNR for each species (row) and each set of orthologous genes (column). Orange and yellow indicate respectively moderate and strong match to the DNA target sequence position-weighted matrix. Black indicates that the corresponding species possesses a gene belonging to the corresponding set of orthologs, while grey indicates that the species does not possess an orthologous gene. Arbitrary numbers were given to identify the different sets of orthologous genes (Table 2). The presence of DNR (blue), FixK (green) or FNR (red) in each genome is indicated by a bar on the right side of the heatmap. Genes that were experimentally determined to be *R. sphaeroides* FnrL target genes are indicated by a black box below their labels. Species are organized according to the phylogenetic tree presented in Figure S1.

To assign predicted target genes to FNR, FixK, or DNR and thus, reconstruct their core regulons, we identified all occurrences of the shared DNA target sequence in the promoter regions of genes in each of the 87 genomes (with an estimated false-discovery rate of ∼15% and p-value≤0.001) (Figure 3, Step A). Because bacterial genes are often organized in transcription units, where multiple genes share a common promoter, each identified DNA target sequence from step A was then linked to all the genes within the nearest predicted transcription unit (see Materials and Methods for how we predicted transcription units). Next, we assembled sets of orthologous genes by clustering all genes across the 87 genomes using the same approach that we used to identify the CRP/FNR super-family functional groups. This approach predicted ∼25,000 distinct sets of orthologous genes distributed in the genomes of the 87 α-proteobacterial species we analyzed. Then, we constructed phylogenetic profiles of target genes based on the occurrence of the common DNA-binding sites within each set of orthologous genes (Figure 3, Step B). Finally, to predict the respective regulons of FNR, FixK, and DNR, for every set of orthologs, we calculated the similarity between the phylogenetic profiles of (i) target DNA-binding sites and (ii) FNR, FixK, or DNR (Figure 3, Step C). This approach allowed us to assign sets of target genes to the regulator with which they shared the most similar phylogenetic profile (Figure 3, Step D, Figure 4, and Table 1). To restrict our predictions to the most conserved members of the respective regulons, we set a cut-off to include target genes that had a phylogenetic profile that was at least 20% similar to one of the three regulators. Considering one example of regulon predictions, the predicted target genes (in yellow in Figure 4) of *Loktanella vestfoldensis* were assigned mostly to the FNR regulon (Figure 4), consistent with the fact that *L. vestfoldensis* possesses a FNR-type regulator but no FixK- or DNR-type regulators. In addition, orthologs of *L. vestfoldensis* target genes were also predicted to be FNR target genes in many other α-proteobacterial species. On the other hand, our predictions probably did not reveal the entire FNR regulon of *L. vestfoldensis* since this method only captured target genes that are conserved in at least 20% of the species that possess FNR. From this comparative analysis, we were able to predict, using genomic sequence information only, conserved members of the FNR, FixK, and DNR regulons, even though each of these proteins recognizes a very similar DNA target sequence.

**Table 1:** Gene product annotations of the predicted members of the DNR, FixK, and FNR regulons determined from the comparative genomics analysis of α-proteobacteria.

	ID^1^	Gene product annotation^2^	*Rsph* locus^3^
**DNR**			
	616	50S ribosomal protein L4	RSP1717
	689	50S ribosomal protein L23	RSP1718
	1300	putative universal stress family protein, UspA	RSP3802
	1696	heme- and copper-containing membrane protein, NnrS	RSP0328
	1847	transcription factor, DNR	
*	2800	peptidase U32 family	RSP0465
	2903	respiratory nitrate reductase alpha-subunit	
*	2966	putative lipid carrier protein	RSP0466
	3120	respiratory nitrate reductase beta-subunit	
	4023	respiratory nitrate reductase delta-subunit	
	4488	putative nitrite transporter	
**FixK**			
	114	response regulator receiver protein, FixJ	RSP0907
	321	putative ABC transporter ATP binding protein	RSP1628
	329	putative ABC transporter permease protein	RSP2459
	332	PAS/PAC sensor signal transduction histidine kinase, FixL	RSP0909
	1387	putative ABC transporter periplasmic substrate binding protein	RSP2811
	1551	heat shock protein Hsp20	
	1862	putative HlyD family secretion protein	RSP3160
	2072	putative ABC transporter permease protein	RSP3157
	2238	putative phosphoketolase	
	2256	putative signal transduction protein with CBS domains	
	2518	putative ABC transporter subunit	RSP3159
	2551	cytochrome c class I	
	2589	putative kinase	RSP0470
	2810	hypothetical protein	
	2855	putative xanthine and cobalt dehydrogenase maturation factor	RSP1934
	3100	putative Zinc binding alcohol dehydrogenase	
	3532	hypothetical protein	
	5478	hypothetical protein	
**FNR**			
*	28	putative heavy metal translocating P-type ATPase	RSP0690
*	74	glutamyl-tRNA reductase	RSP2984
*	125	cytochrome c oxidase subunit I	RSP1877
*	129	putative universal stress protein, UspA	RSP0697
*	219	cytochrome c oxidase subunit II	RSP1826
*	301	transcriptional regulator, FNR/FixK	RSP0698
	555	putative dimethyladenosine transferase	RSP2905
	1083	putative peptidase U62 modulator of DNA gyrase	RSP1825
*	1230	oxygen-independent coproporphyrinogen III oxidase	RSP0699
*	1264	iron-sulfur binding protein RdxA/RdxB/FixG family	RSP0692
*	1289	cytochrome c oxidase cbb_3_-type subunit III	RSP0693
*	1331	cytochrome c oxidase cbb_3_-type subunit I	RSP0696
*	1348	cytochrome c oxidase cbb_3_-type subunit III	RSP0695
*	1758	trans-membrane cation transporter, FixH family	RSP0691
*	1774	putative outer membrane protein, OmpW	RSP2507
*	1915	cytochrome oxidase maturation protein cbb_3_-type	RSP0689
*	1987	putative protoporphyrin monomethyl-ester oxidative cyclase	RSP0281
*	2282	cytochrome c oxidase cbb_3_ type subunit IV	RSP0694
	2905	hypothetical protein	
*	3768	putative DnaK suppressor protein	RSP0166

*Genes for which promoter regions have been shown to be bound by FnrL in *R. sphaeroides* in this study.

**^1^**Arbitrary ID numbers given to the sets of orthologous genes determined across the 87 α-proteobacteria.

**^2^**Functional annotation resulting from the consensus of all the annotations of the genes constituting each set of orthologs.

**^3^**Locus ID of *R. sphaeroides* genes if one is present in the sets of orthologs.

### The predicted FNR regulon is the most conserved across α-proteobacteria

Several patterns emerged from the distribution of the predicted regulons (Figure 4). First, the predicted FNR regulon appeared to be more conserved than those for FixK, or DNR across the 87 α-proteobacterial species. The most conserved part of the predicted FNR regulon contained 6 sets of orthologous genes (including genes encoding FNR itself) in about 60% of the genomes. In addition, a predicted FNR regulon of 20 sets of orthologs was present in 27 species of the *Rhodobacterales* order (Figure S1). Conversely, the composition of the predicted FNR regulon split the *Rhizobiales* order into two groups. The first group of *Rhizobiales* (18 species containing *Rhizobium*, *Mezorhizobium*, *Sinorhizobium*, *Agrobacterium* and others) had a fairly well conserved FNR regulon of ∼13 sets of orthologs. In contrast, in the second group of *Rhizobiales* (19 species, containing *Bradyrhizobium*, *Nitrobacter*, *Rhodopseudomonas*, *Methylobacterium* and others), the predicted FNR regulon was significantly reduced or missing. On the other hand, this second group of *Rhizobiales* is predicted to possess a well-conserved FixK regulon (18 sets of orthologs), possibly indicating a greater role of FixK in the anaerobic or low-oxygen lifestyle of these bacteria. Finally, the 11 sets of orthologs in the predicted DNR regulon were not well conserved or consistent within the species phylogeny, suggesting that DNR plays a more limited or a specialized role in gene expression among α-proteobacteria than either FNR or FixK. In summary, for each of the three global transcription factors, our analysis predicted regulon members that are conserved across α-proteobacteria as well as target genes that were found only in a subset of organisms.

### Experimental tests of the predicted FNR regulon using *Rhodobacter sphaeroides* FnrL

**Figure 5:**
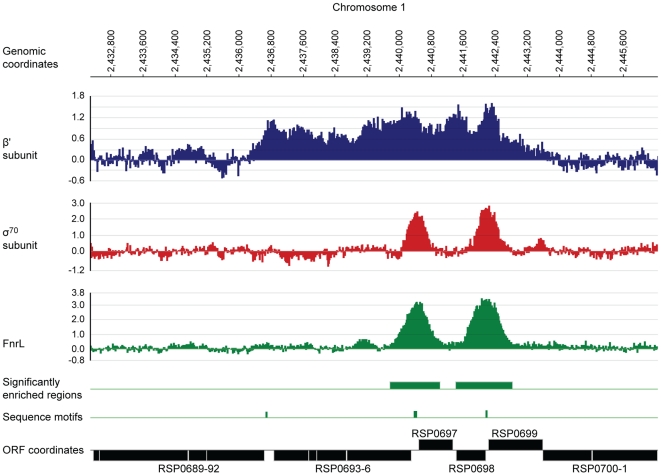
Identification of FnrL binding sites in the *R. sphaeroides* genome by ChIP–chip assays. A representative region of the *R. sphaeroides* genome showing profiles resulting from the enrichment of DNA fragments by immuno-precipitation of the β′ subunit (blue) or σ^70^ (red) subunit of RNA polymerase or FnrL (green) is plotted along the indicated genomic coordinates. The data plot the log_2_ of the ratio of the immunoprecipitated sample to the control sample as a function of probe location along the genome (coordinates are indicated in base pairs). DNA regions significantly enriched (p-value ≤0.01) by FnrL immuno-precipitation (green boxes), positions of sequences matching the FnrL consensus binding site (green tick mark) and the coordinates of annotated genes (black boxes). The data were plotted using SignalMap 1.9 (NimbleGen Systems).

To evaluate our predictions, we directly identified members of the *R. sphaeroides* FnrL regulon using chromatin immuno-precipitation on a chip (ChIP-chip) assays, DNA target sequence analysis, and expression profile clustering. FnrL is a member of the FNR sub-family and contains an O_2_-labile [4Fe-4S] cluster (T. Patschkowski and PJ. Kiley, unpublished data), like its homolog FNR in *E. coli* [4]. To probe genome-wide interactions of FnrL with DNA *in vivo*, we used antibodies to FnrL for ChIP-chip assays [12]. FnrL activity is high in the absence of O_2_ [28], so we analyzed these interactions in wild-type *R. sphaeroides* growing under anaerobic conditions in the presence of light (photosynthetic growth conditions). By identifying regions of the genome that were significantly enriched by immuno-precipitation with antibodies against FnrL (p-value ≤0.01) in three biological replicates, we found 27 sites bound by FnrL (Table 2, Figure 5). Of these 27 sites, 6 were in the promoter regions of genes previously shown to require FnrL for increased activity in the absence of O_2_ [28]–[32], illustrating that this assay identifies bona fide FnrL binding sites.

**Table 2:** Putative FnrL binding sites detected by ChIP–chip analysis or by bioinformatic analysis of the *R. sphaeroides* genome sequence.

Chr^1^	FnrL ChIP-chip peak coordinates^2^		σ^70^ peak^3^	Putative FnrL binding sequence^4^			FnrL target genes^5^		
	Begin	End		Begin	Scores	Sequences	Loci IDs	Regulation	Annotation
Chr 1	408824	409553	+	409223	2156.75	TTGACgcggATCAA	RSP1819-7	+	*feoABC*
Chr 1	417320	418709	+	417975	2501.25	TTGATtcagATCAA	RSP1826-9	−	*coxII-X-XI-III*
Chr 1	476545	477386	−	477119	2554.25	TTGATctggATCAA	RSP1877-6	−	*coxI*
Chr 1	792149	793028	+	792528	2249.50	TTGATacgcATCAA			
Chr 1	862277	863397	+	862812	2276.00	TTGATtcagGTCAA	RSP2247	+	*fusA*
Chr 1	963978	964891	+	964492	1693.00	ATGACgcagATCAA	RSP2337	+	*ccpA1*
Chr 1	1022044	1022949	+	1022541	2368.75	TTGACttagATCAA	RSP2395		*ccpA2*
Chr 1	1152077	1152912	+	1152640	2249.50	TTGACgcagATCAA	RSP2507	+	*ompW*
Chr 1	1217112	1218401	+	1217769	2024.25	TTGACgcagGTCAA	RSP2573	+	
Chr 1	1675600	1676545	+	1676046	2143.50	TTGATccttATCAA	RSP2984	+	*hemA*
Chr 1	1679670	1680230	−	1680004	−996.75	GTGACttagGGCAG			
Chr 1	1811885	1812675	+	1812207	1971.25	CTGATgcagATCAA	RSP0100-12	+	*nuoABCDEFGHJKLMN*
Chr 1	1881897	1882994	+	1882413	2249.50	TTGACctgcATCAA	RSP0166	+	*dksA*
Chr 1	2007383	2008346	+	2007816	2117.00	TTGACatgcATCAA	RSP0281-76	+	*bchEJGP*
Chr 1	2046834	2047877	+	2047244	262.00	TTGCGcaggATCAA	RSP0317	+	*hemN*
Chr 1	2193245	2193765	−	2193494	2382.00	TTGATgcggATCAA			
Chr 1	2201048	2202201	+	2201632	2342.25	TTGATgtagGTCAA	RSP0466-4	+	
							RSP0467-8	+	*ubiD*
Chr 1	2206264	2207340	+	2206759	1613.50	TTGACttcaGTCAA	RSP6116	?	
Chr 1	2439761	2441022	+	2440385	2196.50	ATGATgtcgATCAA	RSP0696-3	+	*ccoNOQP*
				2440417	2196.50	TTGACatggATCAA	RSP0697	+	*uspA*
Chr 1	2441409	2442838	+	2442182	2501.25	TTGATtcagATCAA	RSP0698	−	*fnrL*
							RSP0699	+	*hemZ*
Chr 1	2518226	2519045	+	2518696	1746.00	CTGATctgcGTCAA	RSP0775	+	
Chr 1	2565774	2566660	+	2566094	2382.00	TTGATgcggATCAA	RSP0820	+	
Chr 1	3026608	3027564	+	3027028	1679.75	TTGAGcaagATCAA	RSP1257-4	+	*phbCfabI*
Chr 2	77569	78562	−	78069	1812.25	TTGACgtcaATCAA	RSP3044	+	*dorS*
P002	22017	26368	+	24255	977.50	TTGACagctGTCAA			
P004	1074	2014	−	1088	−877.50	CAGATcgagATGAA			
P004	51099	52291	+	51739	2050.75	CTGATccagATCAA	RSP4201-4	+	
**Additional putative FnrL binding sites identified by sequence analysis**									
Chr 1				403983	1891.75	TTGACcgaaATCAA			
Chr 1				635560	1679.75	ATGATtttcATCAA			
Chr 1				659055	1653.25	TTGACccgcATCAA			
Chr 1				1185086	16930	CTGATcctcATCAA			
Chr 1				1842368	1732.75	ATGATcttcATCAA			
Chr 1				2104074	1640.00	ATGATcctcATCAA			
Chr 1				2436687	1666.50	TTGACttcgGTCAA	RSP0692-89	+	*rdxBHIS*
Chr 1				2760402	1772.50	ATGACccagATCAA			
Chr 2				403914	1640.00	TTGATtgcgATCAA	RSP3341	+	
Chr 2				748494	1640.00	CTGATaaggATCAA	RSP3640-3	+	*exsB*

**^1^**Chromosomes or plasmids.

**^2^**Genomic coordinates of regions of the genome that were significantly enriched by chromatin immuno-precipitation using antibodies against FnrL.

**^3^**Indicates if genomic regions bound by FnrL overlap with regions bound by σ^70^ as determined by chromatin immuno-precipitation using antibodies against σ^70^.

**^4^**Genomic coordinates, scores (log-likelihood ratio), and sequences of putative FnrL binding sites identified using the position-weighted matrix constructed from the conserved DNA target sequence of the FNR-type proteins across α-proteobacteria.

**^5^**Locus number and annotations of the FnrL target genes. The signs indicates whether the transcription of the target operons is increased (+) or decreased (−) by FnrL binding.

To test which FnrL ChIP-chip sites affect gene transcription, we also used ChIP-chip assays to score binding by the major sigma factor, σ^70^, and the β′ subunit of RNA polymerase in the same cultures. This analysis showed that of the 27 regions bound by FnrL, 22 were also bound by σ^70^ (p-value ≤0.01) (Table 2) and the β′ subunit of RNA polymerase, which also extended as expected across the entire length of transcription units, indicating that these genes were actively transcribed under these conditions. The lack of σ^70^ binding in the other 5 of the 27 genomic regions may indicate that FnrL has a negative effect on transcription, possibly by occluding occupancy by σ^70^-containing RNA polymerase. However, it is also possible that a different σ subunit recognizes these promoters, or that no active promoters are located near these regions under our growth conditions. Overall, our analysis shows that FnrL binds DNA under anaerobic conditions *in vivo* and suggests that by this criterion, the FnrL regulon contains at least 27 operons.

To test if the genomic regions bound by FnrL *in vivo* contained the canonical DNA target sequence predicted to be recognized by this protein, TTGAT-N_4_-ATCAA [28]–[31], we used the MAST software to search the corresponding genomic regions [33] for sequences matching the α-proteobacterial FNR DNA target position-weighted matrix we derived (Figure 2B). Of the 27 regions bound by FnrL, 25 contain a close match to the canonical FNR target sequence (log-likelihood score ≥1613.5) (Table 2). The other two sites contain sequences with less similarity to the canonical sequence and may represent lower-affinity FnrL binding sites or ones where FnrL binding is possibly facilitated by another factor. This analysis supports the prediction that *R. sphaeroides* FnrL recognizes a canonical sequence that is very similar to both the one we predicted for the FNR sub-family in α-proteobacteria (Figure 2B) and the motif recognized by *E. coli* FNR [34]. To identify additional potential binding sites that may have been missed in the ChIP-chip experiment, we also searched the entire genome for matches to the FNR DNA target sequence. Using a log-likelihood score ≥1613.5 in order to keep the false-discovery rate ≤10%, we found only 10 additional matches to the target sequence (Table 2). Of these 10 matches, 7 are located within protein coding sequences and three others fall within intergenic regions.

### Transcription profiling identifies additional candidate *R. sphaeroides* FnrL target genes

**Figure 6:**
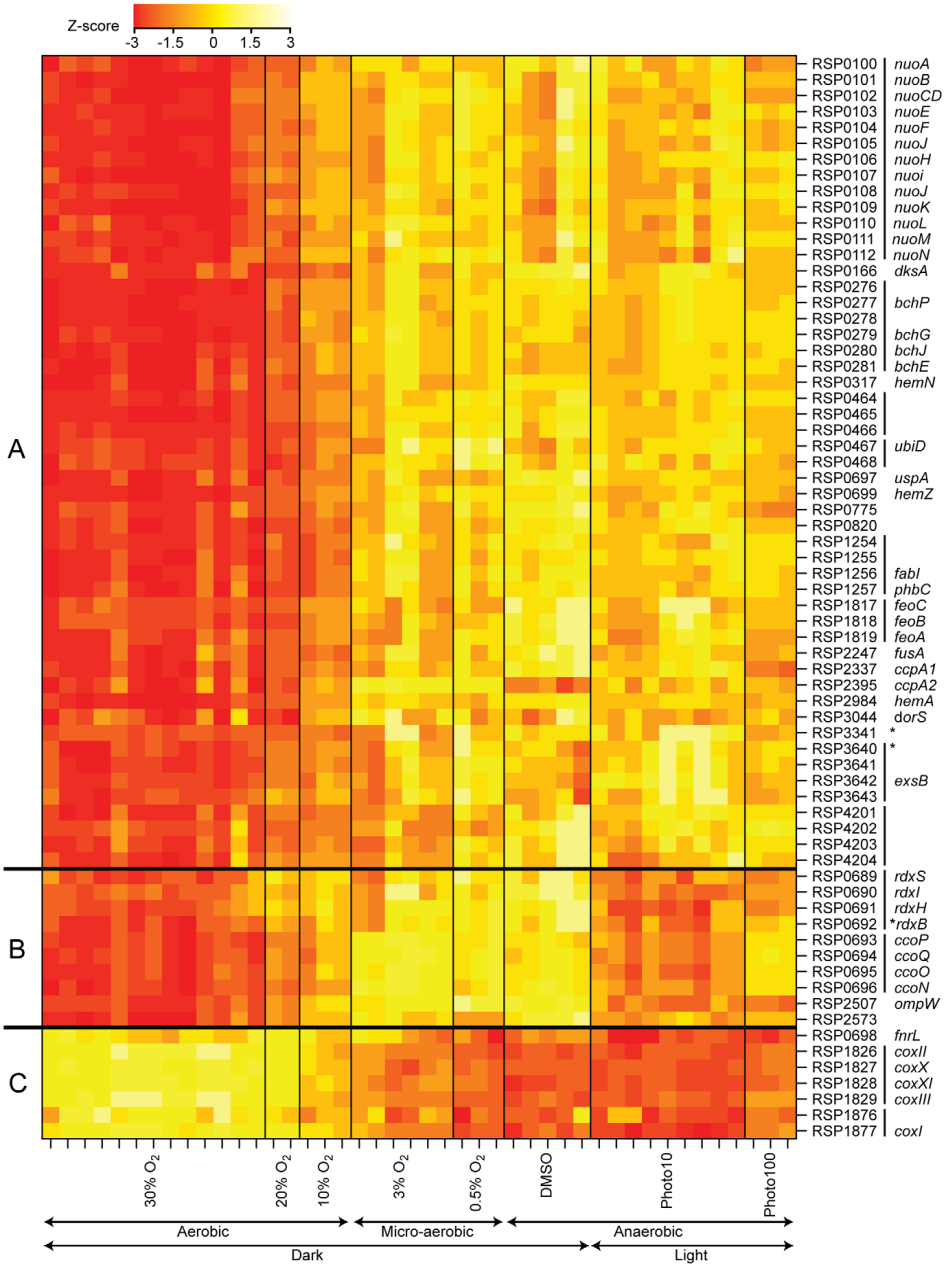
Transcription profile heatmap of members of the FnrL regulon across conditions with varying oxygen tension. The colors represent the relative level of mRNA abundance compared to the mean level of expression for each locus (yellow = high expression, red = low expression). Genes are identified by their locus ID and gene names. Vertical lines next to the locus IDs denote predicted transcription units. Asterisks denote transcription units that had no FnrL ChIP–chip peak detected within their promoter regions but had a sequence matching the FnrL binding site consensus. The amount of oxygen or light in the experimental conditions are indicated below the plot (Photo10 and Photo100 represent illumination of the cultures at 10W/m^2^ and 100W/m^2^, respectively). Genes were grouped according to their expression profiles. Group A contains genes whose expression levels negatively correlate with oxygen tension. Group B contains genes whose expression levels also negatively correlate with oxygen tension but with the exception that these genes have relatively low expression under low light conditions (Photo10). Group C contains genes whose expression levels positively correlate with oxygen tension.

To identify FnrL regulated transcription units, genes within 500 bp on either side of the 37 potential FnrL target sites (27 sites identified by ChIP-chip and the 10 putative FnrL binding sites identified by sequence analysis) were collected and analyzed for O_2_-dependent changes in transcript abundance using publically available global gene expression data from *R. sphaeroides* [14]–[16], [35]. When the transcript abundance profiles were clustered by similarity (Pearson correlation coefficient), the RNA transcript levels of 68 putative FnrL target genes showed O_2_-dependent expression patterns (Figure 6).

One large cluster of co-expressed genes (cluster A in Figure 6) contained 51 protein-coding sequences organized in 20 predicted transcription units. The transcript levels from these 51 genes negatively correlate with culture O_2_ levels, consistent with the hypothesis that FnrL activated their expression. This conclusion is also supported by the co-occupancy of FnrL, σ^70^ and core RNA polymerase at these sites under anaerobic conditions using the ChIP-chip assay. Another cluster of co-expressed genes (cluster B; 10 open reading frames in 4 predicted transcription units) also showed O_2_-dependent changes in RNA abundance. However, cluster B, unlike cluster A, showed less accumulated RNA under anaerobic conditions in the light (the conditions we used to monitor FnrL binding in ChIP-chip assays) than in cells grown anaerobically in the dark. Because FnrL is expected to have the same activity in anaerobic conditions whether light is present or not (as shown by cluster A expression profile in Figure 6), we propose that the transcription of genes in cluster B is affected by an additional, possibly light-responsive factor. Indeed, PpsR/AppA is such a candidate factor since it is known to also control expression of one operon in cluster B (RSP0696-3) in a light- and O_2_-dependent manner [13], . Finally, transcript levels from a third cluster of co-expressed genes (cluster C; 7 open reading frames in three putative transcription units) that were bound by FnrL under anaerobic conditions in the light decreased as culture O_2_ tensions were lowered, so we propose that FnrL directly repressed transcription of these genes.

Overall, our results predict that the 27 transcription units found in gene expression clusters A, B, and C (containing a total of 68 genes) are under direct positive or negative control by FnrL. Twenty-four of these 27 transcription units contained promoter regions bound by FnrL in the ChIP-chip assays (a total of 21 FnrL-bound regions since some of these binding sites were between divergently transcribed operons) (Table 2). The remaining 3 transcription units (RSP0692-89, RSP3341, and RSP3640-3) were associated with a FnrL DNA target sequence (3 of the 10 putative sites detected by sequence analysis) but FnrL-binding was not detected under growth conditions used for ChIP-chip assays. Nevertheless, we propose that these 3 transcription units are FnrL-regulated because of the evidence provided by the gene expression profiling experiments (Table 2). Finally, 6 regions bound by FnrL in the ChIP-chip assays were not associated with any known O_2_ regulated transcription units. These FnrL-occupied sites could represent genomic regions in which FnrL binding did not influence the transcription of neighboring promoters in an O_2_-dependent manner under the conditions explored, ones in which activity of a co-activator is required that is not functional under our growth conditions, or ones in which FnrL controlled expression of transcripts that were not annotated, such as small RNAs.

The predicted function of members within this proposed FnrL regulon is consistent with prior knowledge about the anaerobic lifestyle of *R. sphaeroides*. Functions encoded by members of the FnrL regulon include many components of the electron transport chain. For example, transcription of operons that encode subunits of low-affinity cytochrome c oxidase (RSP1826-29 and RSP1876-77) was apparently repressed by FnrL. In contrast, expression of genes encoding the high-affinity cytochrome *cbb*_3_ oxidase (RSP0693-96), which supports respiration in microaerobic conditions, enzymes for ubiquinone synthesis (RSP0467-8), and the membrane-bound NADH oxidase (RSP0100-12) are proposed to be directly activated by FnrL. Other FnrL-activated functions are involved with the anaerobic lifestyle of *R. sphaeroides*, such as tetrapyrrole (RSP0317, RSP0699, and RSP2984), bacteriochlorophyll biosynthesis (RSP0276-81), or transport of ferrous iron (RSP1817-19), which is predominant in the absence of O_2_. Our data also predicts that expression of *dksA* (RSP0166), which encodes a homolog of a global regulator of stable RNA synthesis and several other cellular functions [38]–[41], is a newly identified target for activation by FnrL under anaerobic conditions in *R. sphaeroides*.

### *R. sphaeroides* possesses an extended FNR regulon that is not absolutely conserved in other α-proteobacteria

A comparison of the computational predictions from the comparative genomics analysis and the experimentally determined *R. sphaeroides* FnrL regulon showed that of the 20 sets of orthologs composing the FNR regulon proposed to be conserved across α-proteobacteria (Figure 4), 17 are part of the *R. sphaeroides* FnrL regulon (Figure 6, Table 2). The remaining three sets of orthologs computationally predicted to be in the conserved FNR regulon (#1083, #2905, and #555 in Figure 4) contained two *R. sphaeroides* genes (RSP2905 and RSP1825) that were not part of the FnrL regulon because their transcript levels were not regulated in an O_2_-dependent manner and no FnrL binding was detected in the ChIP-chip experiment. Nevertheless, it cannot be excluded that FnrL regulates these two genes under growth conditions different from those examined in this study. On the other hand, two experimentally confirmed *R. sphaeroides* FnrL target genes (RSP0465 and RSP0466) were assigned to the DNR regulon by our computational analysis (Figure 4, Table 1). Since *R. sphaeroides* does not possess a DNR ortholog, these two genes may have been acquired through horizontal gene transfer and placed under the control of FnrL. Overall, the agreement between the *R. sphaeroides* FnrL regulon based on experimental and comparative genomic analyses illustrates the utility of the computational methods in correctly predicting target genes for transcription factors.

**Figure 7:**
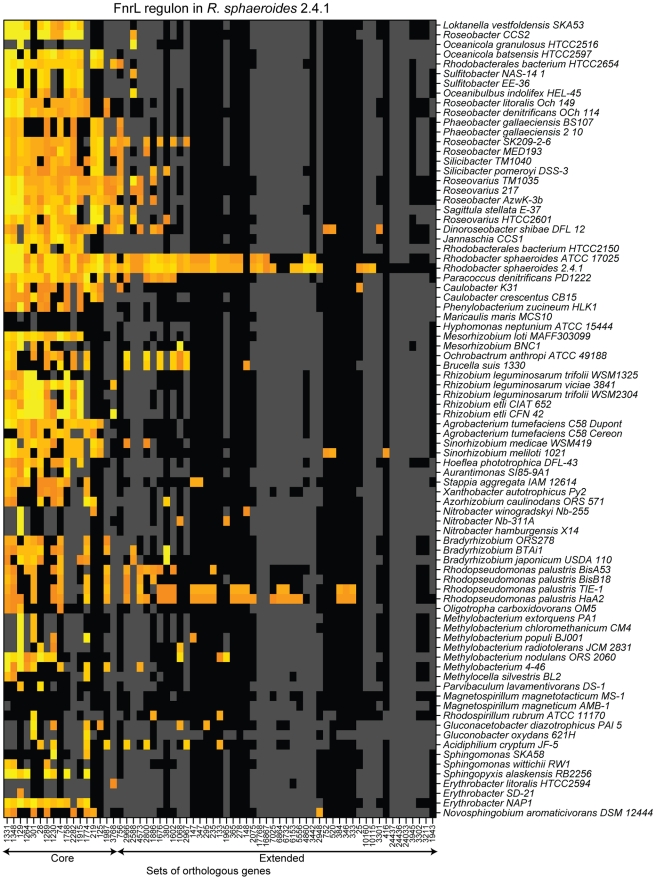
The predicted conservation of the FnrL regulon determined in *R. sphaeroides* across α-proteobacteria. Orange and yellow indicate respectively moderate and strong match to the DNA target sequence position-weighted matrix. Black indicates that the corresponding species possesses a gene belonging to the corresponding set of orthologs, while grey indicates that the species does not possess an orthologous gene. Sets of orthologous genes are labeled with arbitrary numbers. The core FNR regulon, as determined in Figure 4, and the extended FnrL regulon, determined in *R. sphaeroides*, are indicated by arrows below the sets of ortholog labels. Species are organized according to the phylogenetic tree presented in Figure S1.

Nevertheless, the size of the experimentally determined *R. sphaeroides* FnrL regulon (68 genes) is larger than the one proposed to be conserved across α-proteobacteria (20 sets of orthologs); leaving us without information about the regulation of ∼50 predicted FnrL target genes in other α-proteobacteria. Our comparative genomics analysis selected only target genes that were conserved in at least in 20% of the species possessing FNR orthologs. Therefore, to examine to what extent the additional ∼50 genes of the *R. sphaeroides* FnrL regulon were conserved within the 87 α-proteobacteria, we identified the sets of orthologous genes among these bacteria that corresponded to the FnrL target genes and determined which of their promoters contained a predicted FNR DNA target sequence. The results of this analysis indicated that very few of the other α-proteobacteria have FNR target genes in common with *R. sphaeroides* beyond the 20 conserved sets of orthologs (Figure 7, Table S2). As expected, the predicted FNR regulon of another *R. sphaeroides* strain (ATCC 17025) overlaps significantly with the FnrL regulon of *R. sphaeroides* 2.4.1. In addition, only the FNR regulons of the *R. palustris* strains TIE-1 and HaA2, which are photosynthetic bacteria, were predicted to have a significant number of orthologous genes with the extended *R. sphaeroides* FnrL regulon. Interestingly, the predicted overlap of the FnrL regulons between *R. sphaeroides* strain 2.4.1 and *R. palustris* strains TIE-1 and HaA2 is larger than the overlap between *R. sphaeroides* and more closely related species of the *Rhodobacterales* order.

In summary, 17 of the 68 experimentally determined members of the *R. sphaeroides* FnrL regulon were also predicted to be members of a conserved or core FNR regulon across α-proteobacteria. Our data also indicated that *R. sphaeroides* FnrL controls expression of additional genes in a so-called extended regulon that is not always shared with either other purple non-sulfur α-proteobacteria or other closely related species. Overall, our analysis of FNR target genes across the α-proteobacteria indicated that the regulon of orthologous regulators can vary dramatically over a relatively short evolutionary time.

## Discussion

In this work, we combined computational and experimental approaches to infer the evolutionary history of several transcriptional regulatory networks that are conserved across α-proteobacteria. We focused on three transcription regulators, FNR, FixK, and DNR, from the relatively well-characterized CRP/FNR family [3], which control distinct aspects of the anaerobic life-styles of *α*-proteobacteria. We chose to analyze these proteins since they are conserved across a wide range of organisms, they are known or predicted to control critical processes in response to O_2_ deprivation, and they have features that provide challenges to many computational or experimental approaches. Our characterization of the *R. sphaeroides* FnrL regulon and published work (see below) supports that our method correctly predicted many conserved target genes for all three of these transcription factors. This illustrates the utility of this approach even when the proteins have significant amino acid sequence similarity and recognize similar DNA binding sites. In addition, our findings allowed us to propose a hypothesis on the evolution of transcriptional regulatory networks across organisms.

### Experimental support for target gene predictions of individual transcriptional regulatory networks

By determining the similarity between the phylogenetic profiles of each regulator and potential target genes across genomes, we were able to assign target genes to one of the three regulators. In many cases, these assignments were supported by either prior knowledge or experimental data provided in this study. For example, 17 of the 19 predicted members of the core FNR regulon were shown to be direct FnrL targets under the conditions tested. For the *B. japonicum* FixK regulon, expression of blr6070, blr6071, blr4637, blr0497 and blr6074 (ID 3100, 2589, 1551, 5478 and 2256) was shown to depend on FixK_2_ (a FixK-type regulator) [8], [42], as we predicted. We also correctly predicted that expression of RPA4249, RSP4237, RPA4238, RPA4236, RPA1673, RPA4235, RPA1672 and RPA4239 (ID 114, 321, 329, 1387, 2238, 2551, 2810 and 3532) would be dependent on FixK in *R. palustris* [43]. The roles of FixK or FNR in controlling expression of other members of the predicted regulons remain to be tested, but based on our data we expect that many of these candidates will be direct target genes of these transcription factors in α-proteobacteria. Unfortunately, no experimental analysis of the DNR regulon is available in any α-proteobacterium. However, the annotation of the putative target genes of DNR, which is a known nitric oxide sensor, indicates that the predicted role of several members of this regulon is in denitrification, which produces nitric oxide as an intermediate (ID 1696, 2903, 3120, 4023 and 4488).

### Evidence for atypical regulon structures

Because our approach assigned target genes to regulators based on correlations rather than absolute concordance of their respective phylogenetic occurrence profiles, it captured the general patterns emerging from the evolutionary histories of the regulons instead of the exact composition of each regulon in every species. Consequently, regulons were occasionally predicted to exist in species that did not possess the corresponding regulator. For example, we predicted a *Caulobacter* FNR regulon even though these bacteria lack a gene encoding a FNR-type regulator. To explain this observation, we propose that *Caulobacter* species once contained a FNR-type regulator that was displaced by a FixK-type regulator, which now controls expression of these target genes in an O_2_-regulated manner. Indeed, some of these *Caulobacter crescentus* genes are known to require FixK and the O_2_-sensing two-component histidine kinase and regulator, FixLJ for their expression [44].

On the other hand, we also predict that some species possess regulators but lack members of the corresponding core regulon. To explain this observation, we propose that the functions of these orphan regulators have diverged sufficiently to regulate completely different sets of target genes and may actually respond to signals different from those that control activity of FNR, FixK, or DNR. Therefore, these orphan regulators may not actually be orthologs of FNR, FixK, or DNR. Additional experiments are needed to test these hypotheses.

### Potential for regulation of genes by multiple regulators

Because FNR, FixK, and DNR recognize very similar target DNA sequences, cells containing multiple regulators may have target genes that belong to more than one regulon. For example, FNR and FixK are likely to have overlapping regulons because both FNR and FixK activity is regulated by O_2_ [2]. Indeed, 5 members of the known *B. japonicum* FixK regulon (ID 1331, 1264, 1230, 1774 and 129 in Table 1) and 9 genes from the characterized *R. palustris* FixK regulon (ID 74, 129, 301, 1230, 1264, 1289, 1331, 1348 and 1987 in Table 1) [8], [43] are part of our core FNR regulon. These results indicate that, at least in these two species, significant overlap between the FNR and FixK regulons is tolerated. They also illustrate that our computational approach was able to capture the potential overlap between regulators and target genes.

In contrast, there might be less overlap between members of the DNR and either the FNR or FixK, regulons. Indeed, in *Paracoccus denitrificans*, which possesses both a FNR- and a DNR-type regulator (named FnrP and NNR respectively) [45], each protein regulates discrete sets of target genes even though the respective DNA target sequences for these two proteins are very similar. To explain the absence of regulon overlap between FnrP and NNR, Van Spanning *et al.* proposed that other proteins or subtle differences in the DNA binding site play a role in target gene discrimination [45]. Even though the underlying mechanisms for discrimination are unknown, we did not predict significant overlap between the core DNR regulon and those for FNR or FixK. Thus, the phylogenetic relationship between regulators and target genes was able to compensate for missing information about differences between the target sequences of related transcription factors.

Together, these results demonstrate that phylogenetic profiles of regulators and potential target genes can be used successfully to predict the members, function and potential overlap of transcriptional regulatory networks. One benefit of our approach is its ability to decipher relationships between regulators and target genes despite possible overlap in regulon structure which may occur because of similarities in DNA binding sites. Consequently, our assignments provide testable hypotheses about the architecture and role of FNR, FixK, and DNR across α-proteobacteria.

### Distribution and composition of individual transcriptional regulatory networks across species

Our analysis predicts that FNR is the most widely distributed of these three transcription factors we analyzed, since it is found in the genome of 87 α-proteobacteria (Figure 1). For the most part, the genes found in a predicted core α-proteobacterial FNR regulon encode enzymes for micro-aerobic or anaerobic respiratory growth, including synthesis of heme (ID 1230 and 74) and the high-affinity cytochrome *cbb*_3_-type oxidase (ID 1289, 1331, 1348, 1915 and 2282) (Table 2). Other genes in the core α-proteobacterial FNR regulon were predicted to encode metal cation transporters (ID 28 and 1758) that are required for activity of cytochrome *cbb*_3_-type oxidase in *B. japonicum* and *R. sphaeroides* [46], [47]. Since the cytochrome *cbb*_3_-type oxidase contains a copper cluster, it was proposed that these putative transporters maintain cellular copper homeostasis [48]. The *ompW* gene was also predicted to be part of the core α-proteobacterial FNR regulon and its expression was decreased under aerobic conditions in *R. sphaeroides*. In *Salmonella enterica*, OmpW mediates transport of methyl viologen (paraquat) [49], a compound which can generate reactive oxygen species under aerobic conditions [50]. Thus, it is possible that reducing OmpW protein levels in α-proteobacteria in response to increases in O_2_ tension also helps reduce damage from reactive oxygen species, possibly by preventing the uptake of redox mediators. Another member of the predicted core α-proteobacterial FNR regulon is *uspA*, a universal stress family protein, which is involved in stress resistance [51] and required for survival during energy starvation under anaerobic conditions in *Pseudomonas aeruginosa* [52].

In contrast to the wide distribution of FNR homologs, another CRP/FNR family member, FixK, is generally restricted to α-proteobacteria in the *Rhizobiales* order. The predicted core FixK regulon was best defined in the *Bradyrhizobiaceae* family (*Nitrobacter*, *Bradyrhizobium*, and *Rhodopseudomonas*), where it includes genes encoding sensing components of the O_2_-responsive signal transduction cascade, FixLJ (ID 332 and 114) [8], [42]. Moreover, species in the *Bradyrhizobiaceae* family that have a well-defined FixK regulon, also have only 7 genes predicted to be part of the core conserved FNR regulon. Another characteristic of the FixK family correlating with this observation is provided by the recent finding that *B. japonicum* FixK_2_ activity can be regulated by modification of a cysteine residue in response to oxidative stress [53]. Indeed, a protein sequence alignment of all the FixK orthologs analyzed in this study revealed that this particular cysteine residue is conserved only in *Nitrobacter*, *Bradyrhizobium*, and *Rhodopseudomonas* species, indicating that FixK may have a specialized role in these species when compared to the FixK family members in the rest of the α-proteobacteria. Taken together, we propose that an extended FixK regulon, a reduced FNR regulon, and the presence of a conserved reactive cysteine residue in some FixK proteins is part of an evolutionary transition in which FixK acquired some FNR target genes and other functions to integrate them into the lifestyle of *Bradyrhizobiaceae* species. Indeed, it appears that FixK-type regulators diverged from the FNR-type group only very recently [3], probably as an adaptation to a specific ecological niche or signal encountered in their environments. On the other hand, this model suggests that the FixK orthologs present in α-proteobacterial species other than *Nitrobacter*, *Bradyrhizobium*, or *Rhodopseudomonas*, do not share a common set of target genes and consequently have unknown roles in the transcriptional regulatory networks of these bacteria.

The results of our analysis also predicted that members of the DNR regulon include genes known to be involved in nitrate respiration, the first step in denitrification (ID 1696, 2903, 3120, 4023 and 4488). Previous work indicated that NnrR, another member of the CRP/FNR super-family that is involved in NO-dependent regulation [54], is responsible for controlling denitrification in α-proteobacteria [55]. Thus, the small size of the predicted DNR regulon members may indicate a more limited role of this regulatory network in α-proteobacteria in favor of NnrR. This is an interesting hypothesis because members of the NnrR family have a predicted DNA target sequence less similar than DNR does to FNR and FixK (Figure 2). Therefore, NnrR orthologs provide an alternative to DNR to regulate functions in response to nitric oxide and resolve potential cross talk among the different regulatory networks.

### Evolution of individual regulatory networks across α-proteobacteria

Our results provide support the hypothesis that bacterial transcriptional networks are often composed of a core set of genes that is widely conserved across related species, and a larger variable gene set that is specific to a smaller number of species [12], [55]–[59]. For example, we predicted that the core FNR regulon (about 20 genes, Table 2) contains genes involved in the response to O_2_ deprivation, which is a conserved function for this protein across many bacteria [3]. In contrast, the predicted extended *R. sphaeroides* FnrL regulon (another 48 genes, Table 1) mostly encodes functions involved in photosynthetic metabolism, a specialized anaerobic lifestyle for this organism. Moreover, we predicted that species closely related to *R. sphaeroides* and that are proposed to have a photosynthetic lifestyle, such as *Jannaschia* CCS1 or *Dinoroseobacter shibae*, do not share more than one third of the FnrL regulon with *R. sphaeroides* (Figure 7). Such observations suggest that, over a relatively short evolutionary time scale, the composition of the FnrL regulon changed significantly.

### Do environmental factors drive regulatory network evolution?

We propose that the placement of O_2_-dependent and photosynthetic functions within the *R. sphaeroides* FnrL regulon was a result of adaptation to correlated changes in light and O_2_ availability that this organism encounters in nature. Support for the relationship between environmental factors and the composition of regulons is also found in the role of FixK, which controls both the symbiotic relationship between *B. japonicum* with soybean [8], and functions involved in O_2_ utilization [20]. For this plant symbiont, it appears that O_2_ limitation is associated with establishment of root nodules on its host plant. Another example of adaptation to correlated changes in the environment was seen in *E. coli*, which has coupled its transcriptional responses to temperature and O_2_ fluctuations to mirror the co-variation of these two factors when the bacterium travels from the open environment to the gastrointestinal tract [61]. Furthermore, this regulatory connection was rapidly lost when *E. coli* was exposed to an environment where temperature and O_2_ varied independently [61]. Such associative learning may be a widespread mechanism that provides a selective advantage during adaptation to new environmental niches. Presented with a new set of conditions, cell survival depends on the appropriate response to environmental changes. Therefore, if any new environmental signals correlate with other signals that can already be sensed by the cell, genetic changes that link appropriate target genes to an existing regulator would give the cell a competitive advantage. Such rewiring or expansion of regulatory networks may occur more frequently than independent evolution of a sensor, regulator and promoter elements, because of the high-rate of bacterial gene transfer and recombination. The promoter elements necessary for the expansion of transcriptional networks (i.e. binding sequences) can be found in the conserved core regulon, which defines a compact functional unit. Indeed, the conserved core regulon often contains the sensor/regulator itself and proteins directly relevant to the primary signal sensed. Moreover, genes of the core regulon are often physically co-located on the genomes. For example, in many species of α-proteobacteria, the structural gene for FNR is found in the immediate genomic neighborhood of its target genes that encodes for the cytochrome c oxidase cbb_3_-type and accessory proteins.

This model for the evolution of bacterial transcription regulatory networks is consistent with previous analyses [62], [63]. Babu *et al.* concluded that the structure of transcriptional regulatory networks evolves faster than target genes and metabolic networks and that inhabitants of similar ecological niches are more likely to share conserved regulatory networks even if they span wide phylogenetic distances. [64]. These observations support the view that large portions of a so-called extended regulon can be determined by environmental conditions. It remains to be determined if the composition of the core and extended regulons evolve on different time scales.

In summary, this work demonstrates the utility of combining computational and high-throughput experimental approaches to define the composition, function and evolution of regulatory networks. Our approach predictions the target gene composition of these networks even in cells that possess multiple DNA-binding proteins that recognize very similar DNA target sequences. Thus, we expect our approach will be useful to similar analyses of other transcriptional regulatory networks if the DNA binding sites of regulators are known or can be predicted. By studying transcriptional regulators that are critical to a low O_2_ or anaerobic lifestyle, we were also able to identify new physiological functions associated with these regulators. Finally, our results support a model for the evolution of transcriptional regulatory networks. In this hypothesis, the core conserved elements, comprising the transcription factor, target genes and promoter elements represent a ‘start-up kit’ containing elements available to expand the regulon according to factors encountered that are correlated in nature.

## Materials and Methods

### Strains, media, and growth conditions

*R. sphaeroides* 2.4.1 strain was grown in Sistrom's succinate-based minimal medium A [65] at 30°C in 500 ml cultures. To maintain anaerobic photosynthetic conditions the cultures were bubbled with a gas mixture containing 95% N_2_ and 5% CO_2_ and illuminated at a light intensity of 10W/m^2^.

### Chromatin immuno-precipitation on a chip

*R. sphaeroides* cells were harvested at mid-exponential growth phase (∼2×10^8^ colony-forming units/ml) to prepare samples for a ChIP-chip assay [12]. FnrL, the β′ and σ^70^ subunits of RNA polymerase were separately immuno-precipitated with anti-*R. sphaeroides* FnrL rabbit serum, anti-*E. coli* β′ rabbit serum, or 2G10 anti-σ^70^ monoclonal antibodies, respectively. Labeled DNA was hybridized on a custom-made tiling microarray, synthesized by NimbleGen (Roche NimbleGen Inc, Madison, WI), covering *R. sphaeroides* 2.4.1 [12]. Before data analysis, dye intensity bias and array-to-array absolute intensity variations were corrected using quantile normalization across replicates (*limma* package in the R environment) [66]. The log_2_ of the ratio of experimental (Cy3) to control signals (Cy5) was calculated. The data from the biological replicates were averaged for visualization in SignalMap 1.9 software (Roche NimbleGen Inc, Madison WI). Regions of the genome enriched for occupancy by FnrL were identified using TAMALPAIS at p≤0.01 for a threshold set at the 95th percentile of the log_2_ ratio for each chip [67]. Only regions that were significantly enriched in all three replicates were considered. The ChIP-chip data have been deposited in NCBI's Gene Expression Omnibus [68] and are accessible through GEO Series accession number GSE22027 (http://www.ncbi.nlm.nih.gov/geo/query/acc.cgi?acc=GSE22027).

### Microarray gene expression data

To identify genes that show expression patterns correlated with environmental O_2_ levels. *R. sphaeroides* transcription profiling experiment datasets were collected from the Gene Expression Omnibus database (http://www.ncbi.nlm.nih.gov/geo/, platform number: GPL162). The datasets contain gene expression levels from 44 Genechip Custom Express microarrays (Affymetrix, Santa Clara, CA) obtained from the wild-type 2.4.1 strain grown in a succinate-based minimal medium (GSM1620, GSM1671, GSM8108, GSM2410, GSM2421, GSM2422, GSM2423, GSM3030, GSM3031, GSM3032, GSM38777, GSM38778, GSM38779, GSM26242, GSM26243, GSM26244, GSM25295, GSM25296, GSM25297, GSM1672, GSM1673, GSM2425, GSM2426,GSM38780, GSM38781, GSM27348, GSM27349, GSM27350, GSM2418, GSM2419, GSM8109, GSM2429, GSM2430, 2416, GSM2417, GSM8107, GSM3258, GSM3260, GSM3262, GSM38774, GSM38775, GSM38776, GSM3272, GSM3273, GSM3274) [14]–[16]. Expression microarray data were normalized using the RMAexpress v1.0 software (http://rmaexpress.bmbolstad.com/) with background adjustment and quantile normalization [66]. The clustering analysis was done in the R statistical software environment (http://www.r-project.org/) using the Pearson correlation coefficient as a distance between expression patterns and ‘complete’ linkage to construct the cluster hierarchy.

### Determination of sets of orthologous genes

The method adopted to determine sets of putative orthologous proteins was adapted from Li *et al.* [17] with some modifications. First, similarities between all translated protein coding sequences across all tested genomes were discovered using BLASTP algorithm with a cutoff at E-value ≤1e-5 [69]. Each similarity score was normalized by dividing the bit score between two sequences by the maximum of the bit score of each sequence when scored against itself (*norm_score(x,y) = bit_sore(x,y)/max(bit_score(x,x),bit_score(y,y))*). Then, to correct for the fact that the normalized score distributions are dependent on the phylogenetic distance between organisms, all normalized similarity scores between protein sequences of two organisms were divided by the value at the 98^th^ percentile of the distribution of these scores. Sets of related and putatively orthologous proteins were obtained using the MCL 06-058-2 algorithm with settings other than ‘inflation’ set to default [70] (http://www.micans.org/mcl/). Several values for the ‘inflation’ parameter were used to explore the hierarchy of the relationship between sets of proteins. Ultimately, the ‘inflation’ parameter was set to 3.0 to obtain protein sets used in the remaining analysis.

### Species phylogeny reconstruction

The species maximum likelihood phylogeny was constructed using the aLRT-PhyML algorithm [71], [72] (http://atgc.lirmm.fr/phyml/) with default parameters and *E. coli* genome sequences as an out-group. The protein sequence alignment used to reconstruct the phylogeny was derived from 42 sets of orthologs that have only one member in each species. Each protein set was aligned with MUSCLE 3.7 [73] (http://www.drive5.com/muscle/) independently and then all the alignments were concatenated. The global alignment was filtered using GBlocks 0.91b [74] (http://molevol.cmima.csic.es/castresana/Gblocks.html) to remove divergent and poorly aligned positions. The resulting alignment consisted of 5921 positions.

### Phylogenetic transcription factor binding-site analysis

The common binding site model used to carry out the phylogenetic transcription factor binding-site analysis was constructed by aligning the conserved palindromic sequence found in the promoter regions of the genes coding for the FNR, FixK, and DNR orthologs across all genomes considered in this study using MEME [75]. A hidden-Markov model of the binding site motif was constructed with HMMER 2.3.2 [76] (http://hmmer.janelia.org/). The promoter regions, represented by the 300 base pair sequence upstream of the transcriptional start site, of all protein-coding sequences were scored against the model. The distribution of scores for each organism was normalized to a standard distribution to eliminate the influence of varying base composition of the background sequences across organisms. Each protein coding sequence is associated with a motif score, which is represented by its standard deviation from the mean of the score distribution. Scores ≥3.0 were labeled as significant. Because bacterial genes can be organized in polycistronic operons, the promoter scores of the first genes of putative operons were propagated to the rest of the genes in the operons. The score of a predicted downstream gene in the operon was calculated by taking the maximum between its own score and the score of the previous gene multiplied by the probability of the two genes being co-transcribed. The operon predictions were obtained from the VIMSS database (http://www.microbesonline.org/operons/) [77]. After grouping gene products in their respective orthologous sets, the presence of significant DNA target sequences associated with each gene forms a Boolean vector across species. The similarity between the occurrence of a particular transcription factor (*A*) and the occurrence of a binding motif (*B*) was calculated using the Jaccard coefficient (*J*_(*A*,*B*)_ = |*A*∩*B*|/|*A*∪*B*|). Target genes were assigned to the transcription factor to which they shared the most similar phylogenetic profile. Target genes which profiles were not at least 20% similar to one of the three regulators were ignored.

## Supporting Information

Figure S1 Maximum likelihood phylogenetic tree of selected α-proteobacteria. Confidence scores at the branching points are represented by the aLRT statistics from the PhyML algorithm. The position of the root of the tree was determined using *E. coli* K12 as an out-group.
(1.82 MB TIF)

Table S1 Classification of FNR/CRP type regulators.
(0.05 MB XLS)

Table S2 Sets of orthologous genes and annotations corresponding to the FnrL regulon in *R. sphaeroides*.
(0.43 MB DOC)
