# Supplementary figures and images for: Reconstruction of the Core and Extended Regulons of Global Transcription Factors
Source: PLoS Genet. 2010 Jul 22;6(7):e1001027. doi: 10.1371/journal.pgen.1001027 (PMC2908626; doi:10.1371/journal.pgen.1001027)

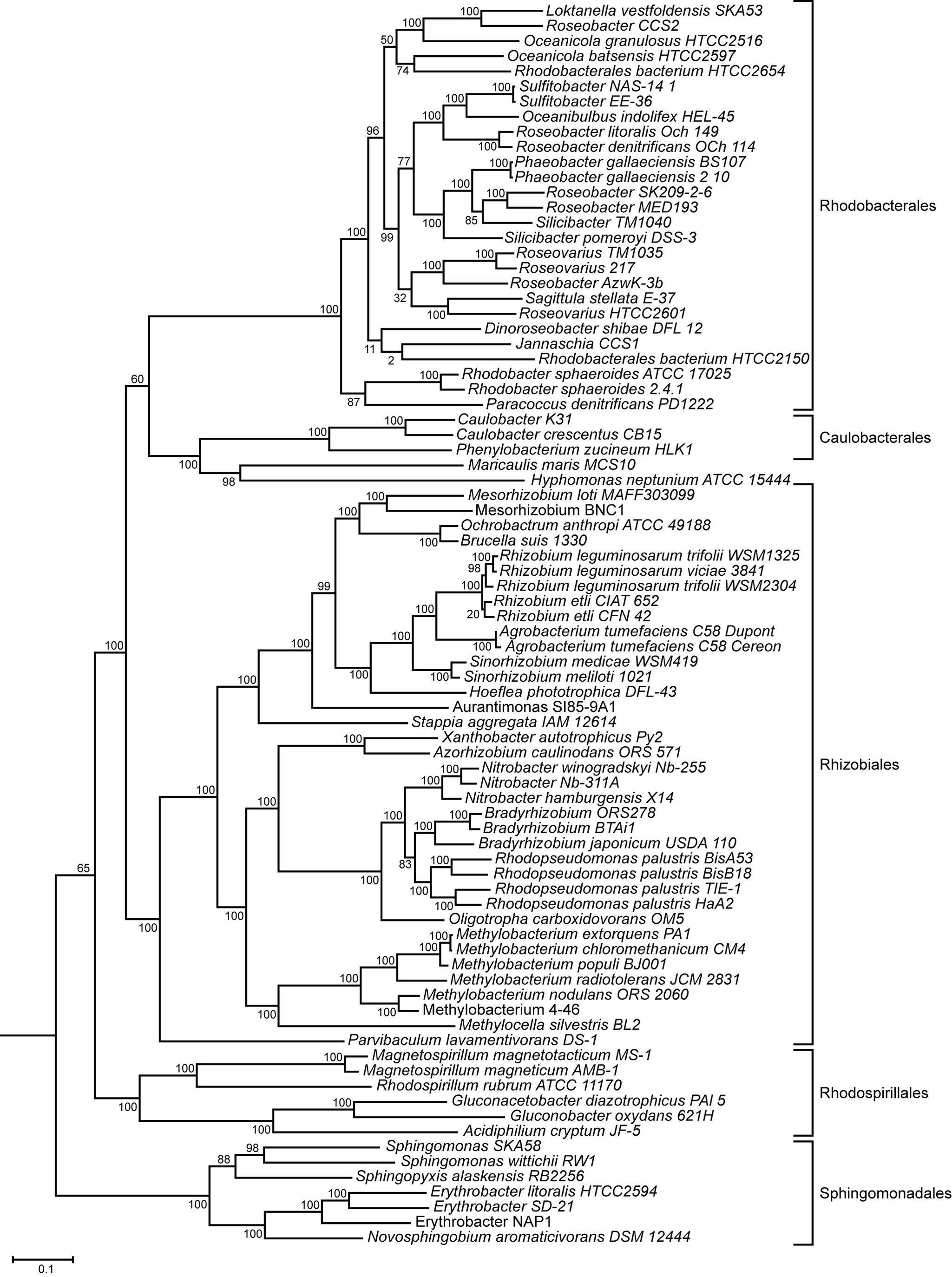

Supplement: Figure S1 — Maximum likelihood phylogenetic tree of selected α-proteobacteria. Confidence scores at the branching points are represented by the aLRT statistics from the PhyML algorithm. The position of the root of the tree was determined using E. coli K12 as an out-group. (1.82 MB TIF) [file pgen.1001027.s001.tif]
