# Supplementary material for: Reconstruction of the Core and Extended Regulons of Global Transcription Factors
Source: PLoS Genet. 2010 Jul 22;6(7):e1001027. doi: 10.1371/journal.pgen.1001027 (PMC2908626; doi:10.1371/journal.pgen.1001027)
Supplement: Table S2 — Sets of orthologous genes and annotations corresponding to the FnrL regulon in R. sphaeroides. (0.43 MB DOC) [file pgen.1001027.s003.doc]

**Table S2. Sets of orthologous genes and annotations corresponding to the experimentally determined FnrL regulon in *R. sphaeroides***.

|  | **ID^1^** | **Locus ID^2^** | **Name** | **Gene product annotation^3^** |
| --- | --- | --- | --- | --- |
|  | 25 | RSP3044 | *dorS* | sensor histidine kinase/response regulator |
| * | 28 | RSP0690 | *rdxI* | putative heavy metal translocating P-type ATPase |
| * | 74 | RSP2984 | *hemA* | glutamul-tRNA reductase |
| * | 125 | RSP1877 | *coxI* | cytochrome C oxidase subunit I |
| * | 129 | RSP0697 | *uspA* | putative universal stress protein, UspA |
|  | 133 | RSP1256 |  | enoyl (acyl carrier protein) reductase |
|  | 147 | RSP0104 | *nuoF* | NADH quinone oxidoreductase F subunit |
|  | 148 | RSP0110 | *nuoL* | NADH dehydrogenase subunit l |
| * | 219 | RSP1826 | *coxII* | cytochrome C oxidase subunit II |
|  | 235 | RSP2247 | *fusA* | elongation factor G |
|  | 278 | RSP0102 | *nuoCD* | NADH dehydrogenase subunit D |
|  | 295 | RSP0101 | *nuoB* | NADH dehydrogenase subunit B |
| * | 301 | RSP0698 | *fnrL* | transcriptional regulator, FnrL |
|  | 333 | RSP0106 | *nuoH* | NADH dehydrogenase subunit H |
|  | 346 | RSP0112 | *nuoN* | NADH dehydrogenase subunit N |
|  | 347 | RSP0105 | *nuoG* | NADH dehydrogenase subunit G |
|  | 365 | RSP0100 | *nuoA* | NADH dehydrogenase subunit A |
|  | 380 | RSP1257 | *phbC* | poly(R) hydroxyalkanoic acid synthase class I |
|  | 384 | RSP0107 | *nuoI* | NADH dehydrogenase subunit I |
|  | 416 | RSP1829 | *coxIII* | cytochrome C oxidase subunit III |
|  | 520 | RSP1827 | *coxX* | cytochrome C oxidase assembly factor |
|  | 752 | RSP1828 | *coxXI* | cytochrome C oxidase assembly protein |
|  | 756 | RSP3341 |  | transcriptional regulator BadM/Rrf2 family |
|  | 1068 | RSP1254 |  | acetate kinase |
| * | 1230 | RSP0317 | *hemN* | oxygen-independent coproporphyrinogen III oxidase |
| * | 1230 | RSP0699 | *hemZ* | oxygen-independent coproporphyrinogen III oxidase |
| * | 1264 | RSP0692 | *rdxB* | iron-sulfur binding protein RdxA/RdxB/FixG family |
| * | 1289 | RSP0693 | *ccoP* | cytochrome C oxidase cbb_3_ type subunit III |
| * | 1331 | RSP0696 | *ccoN* | cytochrome C oxidase cbb_3_ type subunit I |
| * | 1348 | RSP0695 | *ccoO* | cytochrome C oxidase cbb_3_ type subunit II |
|  | 1602 | RSP0468 |  | 3-octaprenyl-4-hydroxybenzoate-carboxy-lyase |
|  | 1676 | RSP0467 | *ubiD* | decarboxylase, UbiD family |
| * | 1758 | RSP0691 | *rdxH* | trans-membrane cation transporter, FixH family |
| * | 1774 | RSP2507 | *ompW* | putative outer membrane protein, OmpW |
|  | 1886 | RSP2395 | *ccpA2* | cytochrome C peroxidase |
| * | 1915 | RSP0689 | *rdxS* | cytochrome C oxidase maturation protein cbb_3_ type |
|  | 1943 | RSP3642 | *exsB* | Putative transcription factor, ExsB family |
|  | 1965 | RSP1255 |  | phosphate acetyltransferase |
| * | 1987 | RSP0281 | *bchE* | putative protoporphyrin monomethyl-ester oxidative cyclase |
|  | 2075 | RSP0103 | *nuoE* | NADH dehydrogenase subunit E |
| * | 2282 | RSP0694 | *ccoQ* | cytochrome C oxidase cbb_3_ type subunit IV |
|  | 2588 | RSP0775 |  | cytochrome C family protein |
| * | 2800 | RSP0465 |  | peptidase U32 family |
|  | 2948 | RSP1818 | *feoB* | ferrous iron transport protein B |
| * | 2966 | RSP0466 |  | putative lipid carrier protein |
|  | 2967 | RSP0464 |  | peptidase U32 family |
|  | 3211 | RSP0277 | *bchP* | geranylgeranyl reductase |
|  | 3301 | RSP0279 | *bchG* | bacteriochlorophyll/chlorophyll A synthase |
|  | 3302 | RSP0278 | *pucC* | putative light harvesting 1 (b870) complex assembly protein, PucC |
|  | 3442 | RSP1876 |  | hypothetical protein |
| * | 3768 | RSP0166 | *dksA* | putative DnaK suppressor protein |
|  | 3945 | RSP0276 |  | isopentenyl diphosphate delta isomerase |
|  | 4573 | RSP0280 | *bchJ* | bacteriochlorophyll synthase, BchJ |
|  | 4860 | RSP0820 |  | cytochrome b561 |
|  | 5556 | RSP4201 |  | transcriptional regulator ArsR family |
|  | 6153 | RSP4202 |  | hypothetical protein |
|  | 6732 | RSP0108 | *nuoJ* | NADH ubiquinone/plastoquinone oxidoreductase |
|  | 6864 | RSP0109 | *nuoK* | NADH ubiquinone oxidoreductase |
|  | 7025 | RSP4203 |  | thiol disulfide isomerase and thioredoxins |
|  | 10115 | RSP1819 | *feoA* | ferrous iron transport protein A |
|  | 10160 | RSP1817 | *feoC* | hypothetical protein |
|  | 16867 | RSP2337 | *ccpA1* | hypothetical protein |
|  | 17768 | RSP2573 |  | hypothetical protein |
|  | 24033 | RSP3641 |  | putative PfkB family carbohydrate kinase |
|  | 24436 | RSP3643 |  | hypothetical protein |
|  | 24437 | RSP3640 |  | hypothetical protein |

*Indicates the genes that are part of the predicted core FNR regulon that is conserved across 87 α-proteobacteria.

^1^Arbitrary number ID given to the sets of orthologous genes determined across the 87 α-proteobacteria.

^2^locus ID of *R. sphaeroides* genes.

^3^Functional annotation resulting from the consensus of all the annotations of the genes constituting each sets of orthologs.
